# Mandibular preservation vs. sacrifice following neoadjuvant immunotherapy in locally advanced oral cancer: a comparative study of surgical and quality-of-life outcomes

**DOI:** 10.3389/fonc.2026.1754661

**Published:** 2026-03-04

**Authors:** Qiongqiong Yu, Zhenjie Guan

**Affiliations:** Department of Oral and Maxillofacial Surgery, The First Affiliated Hospital of Zhengzhou University, Zhengzhou, China

**Keywords:** mandibular preservation, neoadjuvant immunotherapy, oral squamous cell carcinoma, quality of life, surgical de-escalation

## Abstract

**Objective:**

This study compared surgical complication, quality of life (QoL), functional recovery, and oncologic outcomes between mandibular preservation (MP) and mandibular sacrificing (MS) procedures in patients with locally advanced oral squamous cell carcinoma (OSCC) abutting the mandible who achieved a radiologic complete response (rCR) following neoadjuvant immunotherapy (NAT).

**Methods:**

A retrospective cohort study was conducted on 78 patients who achieved a primary site rCR post-NAT. Patients were allocated to an MP cohort (n=42) or an MS cohort (n=36) based on the definitive surgery performed. Primary outcomes were major complications (Clavien-Dindo ≥ III) and longitudinal QoL (EORTC QLQ-C30/H&N35). Secondary outcomes included functional recovery and 3-year oncologic survival.

**Results:**

The MP cohort experienced significantly fewer major complications than the MS cohort (2.4% vs. 19.4%, p=0.013), a finding that held in multivariable analysis (aOR: 3.85, p=0.008). The MP cohort also demonstrated a significantly shorter median hospital stay (9 vs. 16 days, p<0.001), lower rates of gastrostomy dependence at discharge (28.6% vs. 63.9%, p=0.002) and at 3 months (0% vs. 11.1%, p=0.037), and superior QoL scores across multiple domains from 6 months onwards. With a median follow-up of 3 years, there were no significant differences in local (p=0.534), regional (p=0.305), or disease-free survival (p=0.332) between the cohorts.

**Conclusion:**

For select patients with OSCC achieving rCR after NAT, a mandibular preservation strategy is associated with significantly less postoperative complication, improved functional recovery and quality of life, while not compromising short-term oncologic control in this cohort. These findings suggest the feasibility of challenging the paradigm of mandatory mandibular sacrifice in exceptional responders, pending further prospective validation.

## Introduction

Oral squamous cell carcinoma (OSCC) represents a significant global health burden (1). A critical oncologic challenge in managing locally advanced disease is tumor proximity or direct abutment to the mandible. In such scenarios, established surgical principles have traditionally mandated a segmental mandibulectomy to ensure adequate oncologic margins, particularly when the tumor involves or is adherent to the mandibular cortex (2). This radical approach, while aimed at optimizing local control, is physiologically and functionally devastating. The creation of a mandibular discontinuity defect necessitates complex microvascular reconstruction, which is associated with prolonged operative times, significant morbidity, and a high risk of major postoperative complications (3). Furthermore, the functional and aesthetic consequences including profound impairments in mastication, deglutition, speech, and facial contour profoundly diminish health-related quality of life (QoL), often leading to long-term dependency on feeding tubes and tracheostomies (4).

The contemporary landscape of OSCC management has been revolutionized by the advent of immune checkpoint inhibitors (ICI). Neoadjuvant immunotherapy (NAT) has emerged as a promising strategy to downstage tumors, eradicate micrometastatic disease, and potentially enhance long-term survival (5). It has introduced new possibilities for surgical de-escalation. Clinical evidence is accumulating to support this paradigm shift. For instance, trials of NAT have demonstrated high rates of radiologic and pathologic response, which have successfully translated into less extensive surgeries. Specifically, studies have shown that a significant proportion of patients who initially required mandibulectomy were able to undergo preservation surgery after a good response to NAT (6). These de-escalation approaches are consistently associated with tangible benefits, including reduced operative times, lower reliance on complex flap reconstruction, shorter hospital stays, and significantly improved quality of life metrics. Critically, initial comparative analyses have indicated that these functional advantages can be achieved without compromising short- to medium-term oncologic outcomes, such as overall and disease-free survival, when compared to traditional upfront surgery (7–9). A remarkable manifestation of this therapeutic efficacy is the achievement of a rCR at the primary site, which poses a compelling clinical question. However, limitations in the existing literature, such as short follow-up durations and a lack of direct comparison to the standard sequence of NAT followed by planned radical resection, underscore the need for the present investigation of whether it is oncologically safe to deviate from the planned segmental mandibulectomy and pursue a mandibular-preserving procedure?

To address this knowledge gap, we conducted a retrospective cohort study comparing patients with locally advanced OSCC abutting the mandible who achieved an rCR post-NAT and subsequently underwent either mandibular preservation or a traditional mandibular-sacrificing procedure. This study aims to rigorously compare the two cohorts across three fundamental domains: surgical complication, health-related QoL and functional recovery; and short-to-medium-term oncologic efficacy. We hypothesize that in carefully selected patients with a documented rCR, a mandibular preservation strategy can yield significantly improved functional and QoL outcomes without compromising oncologic control compared to a standard mandibular-sacrificing approach.

## Methods

### Study design and patient population

This investigation was designed as a retrospective and comparative cohort study of prospectively collected data. It was conducted following approval from the Institutional Review Boards of The First Affiliated Hospital of Zhengzhou University and in strict accordance with the ethical principles outlined in the Declaration of Helsinki. Written consent was obtained from every patient before enrollment.

The study cohort was identified by screening the electronic medical records of all consecutive adult patients (aged 18 years or older) with a histological confirmation of locally advanced OSCC staged as III-IVB according to the AJCC 8^th^ edition. To be eligible for screening, patients must have been diagnosed between January 1, 2018, and December 31, 2023, and their pre-treatment imaging (CT, MRI, or PET/CT) had to document that the primary tumor was involving or directly abutting the mandibular cortex.

Inclusion in the final analysis was contingent upon meeting all of the following criteria:

1. Received at least two cycles of NAT in combination with chemotherapy.
2. Achieved a rCR at the primary site, with specific attention to the tumor–mandible interface, as assessed on restaging imaging following NAT according to RECIST 1.1 criteria.
3. Underwent definitive surgical resection of the primary tumor along with neck dissection within 8 weeks of completing NAT.
4. Possessed a complete medical record, including detailed operative notes, pathology reports, and adequate follow-up data.

Patients were excluded from the study if any of the following applied:

1. Underwent surgery with palliative intent.
2. Had received prior radiotherapy to the head and neck region.
3. Presented with distant metastases at the time of surgery.
4. Had incomplete medical records or were lost to follow-up within 90 days of their surgical procedure.

### Cohort definitions and group allocation

Based on the definitive surgical procedure performed, patients were allocated into two distinct cohorts for comparative analysis. The Mandibular Preservation (MP) Cohort consisted of patients for whom intraoperative findings, following neoadjuvant therapy, permitted a deviation from the pre-operatively planned mandibulectomy; preservation was strictly defined as the absence of a segmental mandibulectomy and encompassed procedures such as periosteal stripping, marginal resection, or cortical shaving of the mandible. In contrast, the Mandibular Sacrificing (MS) Cohort comprised patients who ultimately underwent a segmental or a large composite marginal mandibulectomy as initially planned, despite the documented rCR; this involved the full-thickness removal of a mandibular segment, creating a discontinuity defect that necessitated complex reconstruction, typically with an osseous or osteocutaneous free flap. The final intraoperative decision to pursue a preservation or sacrificing approach was made by the same attending surgeon (Q.Y.), informed by a combination of factors including the degree of tumor detachment from the bone, the quality of the underlying mandible, and the results of frozen section analysis of the soft tissue margins adjacent to the mandible.

### Data collection and study variables

A comprehensive and standardized data extraction protocol was employed to collect detailed information from the electronic health records of all eligible patients. The collected variables were systematically categorized into several key domains. Baseline and demographic characteristics included age, sex, BMI, comorbidities summarized by the Charlson Comorbidity Index (CCI), smoking pack-year history, alcohol use, primary tumor site and subsite, and clinical TNM stage according to the AJCC 8th edition. Neoadjuvant and surgical treatment details encompassed the specific NAT regimen, the number of cycles administered, the occurrence and grade of any immune-related adverse events (irAEs), and specific surgical procedure details such as the type of mandibular resection, total operation time, estimated intraoperative blood loss, and the method of reconstruction. Furthermore, data were collected on the pathologic assessment, which involved the final surgical margin status, the pathologic tumor stage (ypTNM), and the degree of pathologic response in the primary tumor specimen as evaluated by the College of American Pathologists tumor regression grading system, ranging from Complete Response to Poor or No Response. The presence or absence of perineural invasion and lymphovascular invasion was also documented from the final pathology reports.

### Outcome measures

The study evaluated two primary outcome domains: surgical complication and QoL. Surgical complication was rigorously assessed by the incidence of major postoperative complications occurring within 90 days of the definitive procedure, with severity graded according to the Clavien-Dindo (CD) classification system; a major complication was prospectively defined as CD grade III or higher, indicating a requirement for surgical, endoscopic, or radiological intervention. Specific complications tracked included unplanned return to the operating room, free flap failure, the development of an orocutaneous or pharyngocutaneous fistula, wound dehiscence or major infection requiring surgical intervention, significant postoperative hemorrhage, unplanned intensive care unit (ICU) admission, and mortality. The second primary outcome, health-related quality of life, was measured using the validated European Organization for Research and Treatment of Cancer (EORTC) Quality of Life Questionnaires, specifically the core EORTC QLQ-C30 (version 3.0) and the head and neck-specific EORTC QLQ-H&N35 modules. The questionnaire was required to complete preoperatively, 3 months, 6 months, and 12 months after operation.

Secondary outcomes focused on functional recovery and short-term oncologic efficacy. Functional outcomes were evaluated by measuring the duration of the initial postoperative hospital stay and by determining patient dependency on supportive measures, specifically the presence of a gastrostomy tube and/or tracheostomy at the time of hospital discharge and at the 3-month follow-up visit. Furthermore, short-term oncologic efficacy was measured by calculating local recurrence-free survival (LRFS), defined as the time from surgery to the first recurrence at the primary site; regional recurrence-free survival (RRFS), defined as the time to recurrence in the cervical lymph nodes; and disease-free survival (DFS), defined as the time to any recurrence (local, regional, or distant) or death from any cause, with all three metrics being reported at 3 years postoperatively.

### Statistical analysis

All statistical analyses were performed using IBM SPSS Statistics (version 28.0) and R software, with a two-sided p-value of < 0.05 deemed significant. Continuous variables were presented as mean ± standard deviation or median with interquartile range based on their distribution, while categorical variables were summarized as frequencies and percentages. Initial comparisons of baseline demographic, clinical, and treatment-related characteristics between the MP and MS cohorts were conducted using independent samples t-tests, Mann-Whitney U tests, or Chi-square/Fisher’s exact tests, as appropriate, to identify potential confounding factors for adjustment.

The primary outcomes were analyzed with rigorous methodologies. For surgical complication, the incidence of major complications (Clavien-Dindo grade ≥ III) was compared using the Chi-square test, followed by a multivariable logistic regression model to calculate adjusted odds ratios (aOR). For the longitudinal QoL data, a linear mixed-effects model was employed to analyze the EORTC QLQ-C30 and QLQ-H&N35 scores collected preoperatively and at 3, 6, and 12 months postoperatively. This model included fixed effects for group, time, and their interaction, with a random intercept for each patient, allowing for a robust comparison of QoL trajectories between cohorts over time while handling within-patient correlation and missing data.

Secondary outcomes were compared using Chi-square or Mann-Whitney U tests. LRFS, RRFS, and DFS were estimated using Kaplan-Meier curves and compared with the log-rank test. The association of cohort allocation with survival outcomes was further quantified using univariate and multivariable Cox proportional hazards models to calculate hazard ratios (HR), adjusting for pathologic stage and margin status. A *post-hoc* power analysis was conducted to inform the interpretability of the findings given the final cohort sizes.

## Results

### Baseline data

In total, 78 patients were enrolled. The MP (n=42) and MS (n=36) cohorts were generally comparable in several key areas. No statistically significant differences were observed in demographic factors such as age, sex, and BMI, or in lifestyle factors including smoking history and alcohol use. Comorbidity burden and details of neoadjuvant treatment, including the number of cycles and the incidence of irAEs, were also similar between the two groups. All patients received the same neoadjuvant immunochemotherapy regimen (ICI + cisplatin-based chemotherapy). Accordingly, cortical bone erosion on pre-treatment imaging was more common in the MS cohort (33.3%) than in the MP cohort (16.7%), although this difference did not reach statistical significance (p=0.089). The MS cohort presented with more advanced disease, featuring significantly higher Clinical T Stage (p=0.008), with 50.0% having T4a tumors compared to 23.8% in the MP cohort, and a more advanced Overall Stage Group (p=0.003), including a greater proportion of Stage IVB disease (25.0% vs. 9.5%). Clinical N Stage was the only tumor characteristic that did not differ significantly between the cohorts. All patients achieved a complete pathologic response and a clear margin. (**Supplementary Table 1**).

### Surgical complication

The rate of major complications (Clavien-Dindo grade III or higher) was significantly greater in the MS group (19.4%, 7/36) compared to the MP group (2.4%, 1/42), with a p-value of 0.013. While the overall incidence of specific major complications such as unplanned return to the operating room, orocutaneous fistula, free flap failure, significant postoperative hemorrhage, wound dehiscence/major infection, and unplanned ICU admission was low, all individual instances of these events, except for one case of hemorrhage, occurred exclusively in the MS cohort. However, the differences in the rates of these specific complications did not reach statistical significance individually (**Table 1**).

**Table 1:** Postoperative complications within 90 days.

Complication	Overall (n=78)	MP Cohort (n=42)	MS Cohort (n=36)	p
**Major Complication (Clavien-Dindo ≥ III)**	**8 (10.3%)**	**1 (2.4%)**	**7 (19.4%)**	**0.013**
Specific Major Complications				
Unplanned return to operating room	1 (1.3%)	0 (0.0%)	1 (2.8%)	0.458
Orocutaneous fistula	1 (1.3%)	0 (0.0%)	1 (2.8%)	0.458
Free flap failure	1 (1.3%)	0 (0.0%)	1 (2.8%)	0.458
Wound dehiscence / major infection	1 (1.3%)	0 (0.0%)	1 (2.8%)	0.458
Significant postoperative hemorrhage	3 (3.8%)	1 (2.4%)	2 (5.6%)	0.588
Unplanned ICU admission	1 (1.3%)	0 (0.0%)	1 (2.8%)	0.458

Data are presented as number (percentage). p-values are from Chi-square or Fisher's exact test, as appropriate. Statistically significant values (p < 0.05) are in bold with an asterisk.

MP, Mandibular Preservation; MS, Mandibular Sacrificing; ICU, Intensive Care Unit.

Univariate and multivariable analyses were performed to identify factors associated with the occurrence of major complications. In the univariate analysis, the MS cohort was associated with a significantly higher odds of major complications compared to the MP cohort (OR 3.76, 95% CI 1.42-9.97, p=0.008). A clinical T stage of T4a (vs. T2-T3; OR 2.67, 95% CI 1.05-6.78, p=0.039) were also significant risk factors. Other demographic, lifestyle, tumor, and treatment factors, including age, comorbidities, smoking, and neoadjuvant treatment details, were not significantly associated. When these factors were adjusted for in the multivariable analysis, the MS cohort remained the sole independent and statistically significant predictor of major complications (Adjusted OR 3.85, 95% CI 1.42-10.45, p=0.008). The associations for tumor site and T stage were attenuated and were no longer statistically significant in the adjusted model, indicating that the increased risk they presented in univariate analysis may be confounded by their relationship with the surgical approach (**Table 2**).

**Table 2:** Univariate and multivariable analysis of factors associated with major complications.

Factor	Univariate analysis			Multivariable analysis		
	OR	95% CI	p	Adjusted OR	95% CI	p
Treatment cohort (Ref: MP)						
MS cohort	**3.76**	**1.42-9.97**	**0.008**	**3.85**	**1.42-10.45**	**0.008**
Demographics						
Age (≥60 vs. <60 years)	1.45	0.58-3.65	0.426	1.32	0.50-3.50	0.576
Sex (Male vs. Female)	1.22	0.38-3.91	0.741	1.15	0.34-3.89	0.822
BMI (≥25 vs. <25 kg/m²)	1.10	0.41-2.96	0.851	0.95	0.33-2.76	0.927
CCI (≥3 vs. <3)	1.80	0.71-4.54	0.215	1.55	0.58-4.12	0.381
Lifestyle factors						
Smoking (>10 vs. ≤10 pack-years)	1.52	0.59-3.93	0.390	1.48	0.54-4.07	0.450
Alcohol use (>7 vs. ≤7 units/week)	1.35	0.53-3.41	0.530	1.28	0.48-3.45	0.623
Tumor characteristics						
Clinical T stage (Ref: T2-T3)						
T4a	**2.67**	**1.05-6.78**	**0.039**	2.10	0.78-5.66	0.142
Clinical N stage (N2/3 vs. N0/1)	1.63	0.59-4.52	0.345	1.40	0.48-4.06	0.537
Overall stage (Ref: Stage III)						
Stage IVA	1.55	0.45-5.36	0.490	1.25	0.34-4.58	0.740
Stage IVB	2.78	0.67-11.52	0.160	1.95	0.43-8.79	0.386
Treatment factors						
NAT cycles (>2 vs. 2)	1.33	0.49-3.59	0.576	1.25	0.44-3.55	0.678
irAE (Yes vs. No)	0.79	0.31-1.99	0.614	0.75	0.28-2.00	0.569

CI, Confidence Interval; OR, Odds Ratio; MP, Mandibular Preservation; MS, Mandibular Sacrificing; BMI, Body Mass Index; CCI, Charlson Comorbidity Index; NAT, Neoadjuvant Therapy; irAE, immune-related Adverse Event. Statistically significant values (p < 0.05) are in bold.

### QoL

**Figure 1:**
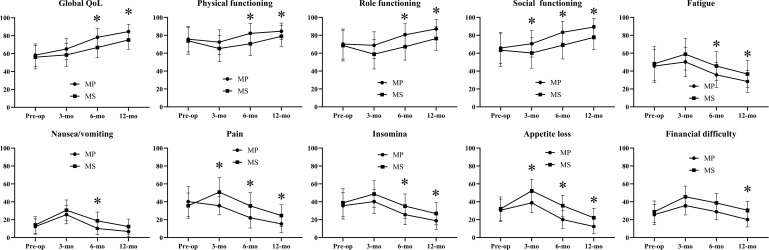
Quality of life in patients assessed using EORTC QLQ-C30: only domains having significant differences indicated by * are presented.

**Figure 2:**
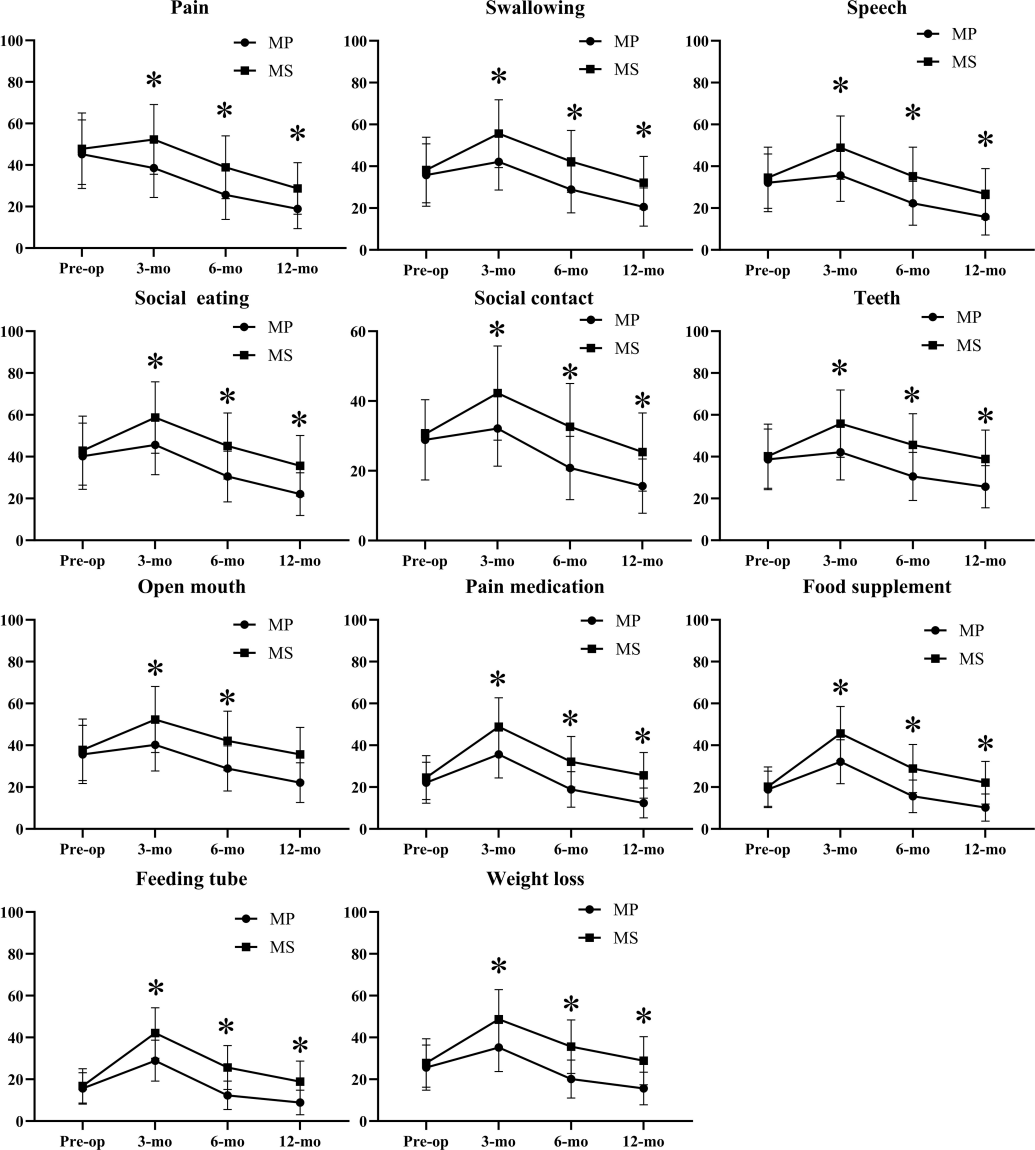
Quality of life in patients assessed using EORTC H&N35: only domains having significant differences indicated by * are presented.

In EORTC QLQ-C30 module (**Figure 1**; **Supplementary Table 2**), while baseline scores were comparable, the MP cohort demonstrated a superior and more rapid improvement across most domains, particularly in Global Quality of Life, Physical Functioning, and Role Functioning, with these differences becoming statistically significant (p < 0.05) by the 6- and 12-month follow-ups. Concurrently, the MP cohort reported significantly lower severity of key symptoms, including pain, fatigue, insomnia, and appetite loss, at these later time points. This pattern indicates that patients who underwent mandibular preservation experienced a substantially lower symptom burden and a faster return to normal daily activities and overall well-being compared to those who required segmental mandibulectomy. These findings were further reinforced by the head and neck-specific EORTC QLQ-H&N35 module (**Figure 2**; **Supplementary Table 3**). The MS cohort consistently reported significantly worse outcomes (p < 0.05) related to swallowing, speech, social eating, and social contact throughout the follow-up period. Notably, domains directly impacted by mandibular integrity, such as problems with teeth and opening the mouth, were also markedly more severe in the MS group. Furthermore, the greater reliance on supportive measures in the MS cohort was reflected in significantly higher scores for pain medication use, nutritional supplements, and feeding tube dependence at the 3-month mark, with these differences persisting, though narrowing, at one year.

### Functional recovery

Significant differences of postoperative functional outcomes were observed between the MP and MS cohorts. The median postoperative hospital stay was markedly shorter for the MP cohort (9 days, IQR 7-12) compared to the MS cohort (16 days, IQR 13-22), a difference that was highly statistically significant (p<0.001). At the time of hospital discharge, functional dependence was substantially more common in the MS group, with a significantly higher proportion of patients dependent on a gastrostomy tube (63.9% vs. 28.6%, p=0.002) and a tracheostomy (55.6% vs. 19.0%, p<0.001). While functional status improved in both groups by the 3-month follow-up, a significant disparity in gastrostomy tube dependence persisted, with 11.1% of MS patients remaining dependent compared to 0% in the MP group (p=0.037). The difference in tracheostomy dependence at 3 months was no longer statistically significant, with only one patient (5.6%) in the MS cohort still dependent. These findings indicate that while mandibular sacrifice may be a necessary oncologic procedure, it is associated with a significantly longer initial recovery period and a higher burden of functional impairment, particularly with regard to swallowing, which persists for a notable subset of patients at three months postoperatively (**Table 3**).

**Table 3:** Postoperative functional outcomes.

Parameter	Overall (n=78)	MP cohort(n=42)	MS cohort(n=36)	p
Hospital stay, days (Median, IQR)	12 (8-18)	9 (7-12)	16 (13-22)	**<0.001**
At hospital discharge				
Gastrostomy tube dependent	35 (44.9%)	12 (28.6%)	23 (63.9%)	**0.002**
Tracheostomy dependent	28 (35.9%)	8 (19.0%)	20 (55.6%)	**<0.001**
At 3-month follow-up				
Gastrostomy tube dependent	4 (5.1%)	0	4 (11.1%)	**0.037**
Tracheostomy dependent	1 (1.3%)	0	1 (5.6%)	0.207

Data are presented as number (percentage) or median (interquartile range). p-values are from Chi-square test for proportions and Mann-Whitney U test for hospital stay. Statistically significant values (p < 0.05) are in bold with an asterisk. MP, Mandibular Preservation; MS, Mandibular Sacrificing; IQR, Interquartile Range.

### Oncologic outcome

**Figure 3:**
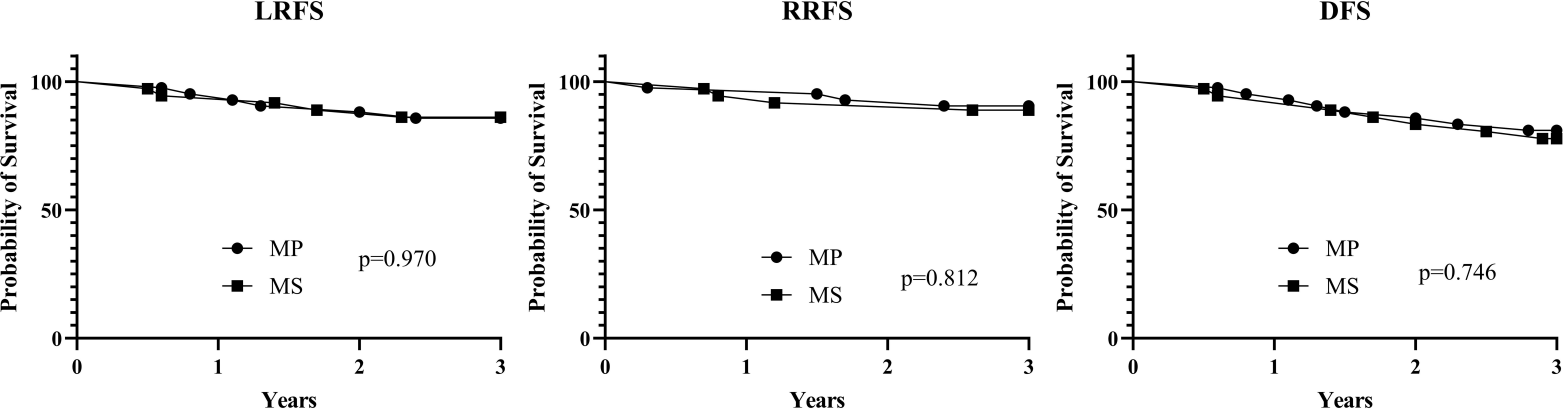
Survival differences in MP and MS cohorts regarding local recurrence free survival (LRFS), regional recurrence free survival (RRFS), and disease free survival (DFS).

No significant detriment was associated with the mandibular preservation approach for LRFS (**Figure 3**). The univariate Cox regression revealed a non-significant HR of 0.51 (95% CI 0.05–4.98; p=0.562) for the MS cohort compared to the MP cohort, indicating no increased risk of local recurrence with preservation. This finding persisted after multivariable adjustment for potential confounders, with an adjusted HR of 0.48 (95% CI 0.05–4.82; p=0.534). This suggests that achieving a rCR at the primary site following neoadjuvant therapy may permit mandibular preservation without compromising local tumor control (**Table 4**).

**Table 4:** Univariate and multivariable cox regression analysis for local recurrence-free survival (LRFS).

Factor	Univariate analysis			Multivariable analysis		
	HR	95% CI	p	Adjusted HR	95% CI	p
Treatment cohort (Ref: MP)						
MS cohort	0.51	0.05 - 4.98	0.562	0.48	0.05 - 4.82	0.534
Age (≥60 vs. <60 years)	1.45	0.24 - 8.69	0.685	1.32	0.21 - 8.18	0.767
Sex (Male vs. Female)	1.12	0.18 - 6.83	0.902	0.95	0.15 - 5.98	0.956
CCI (≥3 vs. <3)	1.80	0.30 - 10.78	0.520	1.65	0.26 - 10.29	0.594
Smoking (>10 vs. ≤10 pack-years)	1.52	0.25 - 9.10	0.649	1.48	0.24 - 9.05	0.671
Clinical T stage (Ref: T2-T3)						
T4a	2.67	0.45 - 15.71	0.279	2.10	0.34 - 12.85	0.417
Clinical N stage (N2/3 vs. N0/1)	1.63	0.27 - 9.71	0.590	1.45	0.24 - 8.69	0.685
NAT cycles (>2 vs. 2)	1.33	0.22 - 7.89	0.756	1.25	0.21 - 7.45	0.806
irAE (Yes vs. No)	0.79	0.13 - 4.70	0.797	0.85	0.14 - 5.12	0.861

CI, Confidence Interval; HR, Hazard Ratio; MP, Mandibular Preservation; MS, Mandibular Sacrificing; CCI, Charlson Comorbidity Index; NAT, Neoadjuvant Therapy; irAE, immune-related Adverse Event.

For RRFS, the surgical cohort was not a significant predictor of outcome (**Figure 3**). The MS cohort showed a non-significant trend toward a higher hazard of regional recurrence in both univariate (HR 1.80, 95% CI 0.53–6.15; p=0.350) and multivariable analyses (Adjusted HR 1.92, 95% CI 0.55–6.76; p=0.305). No other factors, including clinical N stage, or tumor site were significantly associated with RRFS in the models. This implies that the choice of mandibular resection did not independently influence the risk of recurrence in the cervical lymph nodes. (**Supplementary Table 4**).

The composite endpoint of DFS was also comparable between the two cohorts (**Figure 3**). Interestingly, the point estimates for the MS cohort hazard ratio were below 1.0 in both univariate (HR 0.35, 95% CI 0.04–3.18) and multivariable models (Adjusted HR 0.33, 95% CI 0.03–3.12), though these results were highly non-significant (p=0.350 and p=0.332, respectively). Over a median follow-up of 3 years, this analysis provides preliminary evidence that an organ-preservation strategy, when applied to select patients with an excellent response to neoadjuvant therapy, can achieve short-to-medium-term disease control rates that are not inferior to those of traditional, more radical surgery. (**Supplementary Table 5**).

### Stratified analysis by cT stage

**Figure 4:**
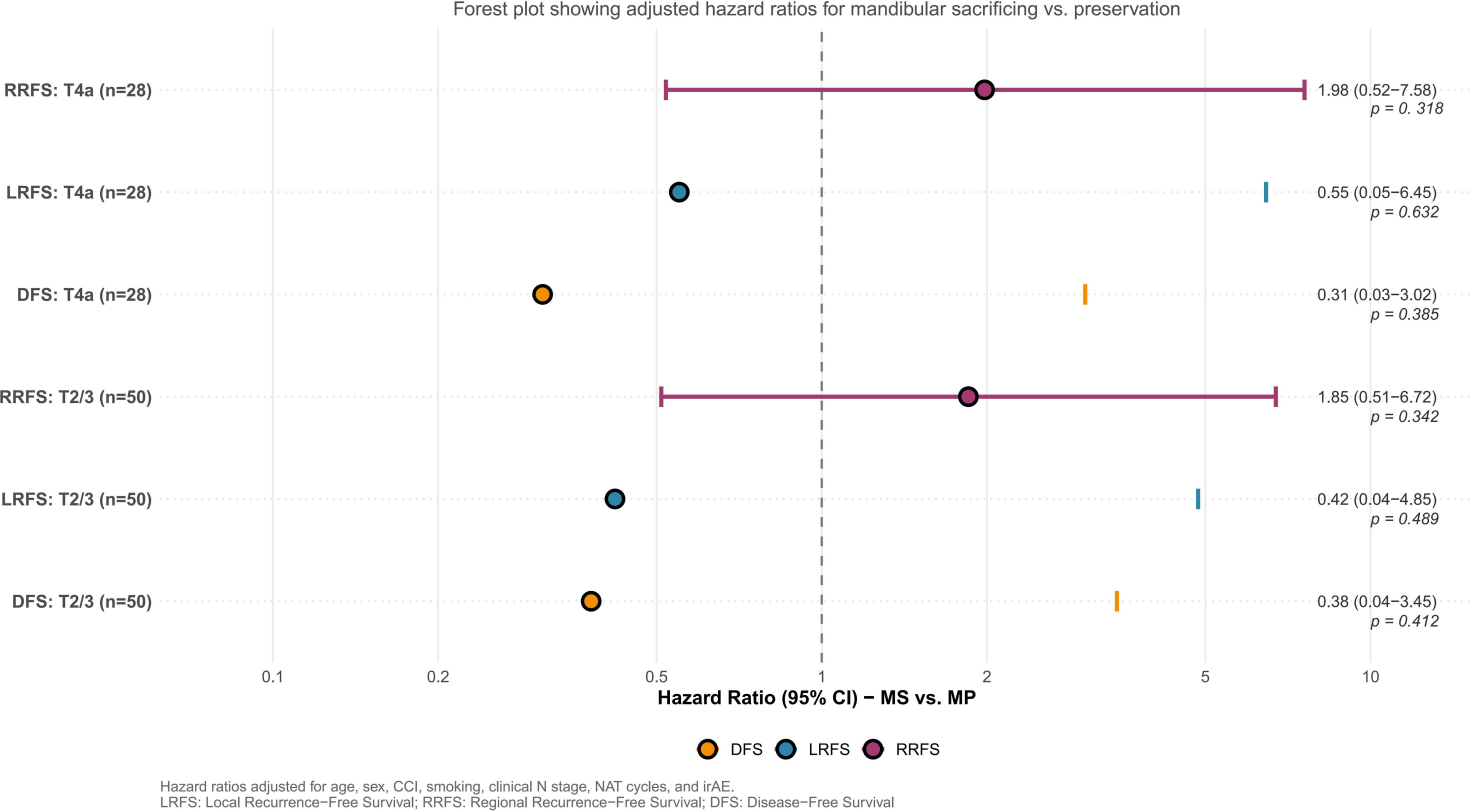
Forest plot of stratified analyses for survival outcomes by clinical T stage.

To address potential confounding from the uneven distribution of baseline T stage, subgroup analyses stratified by clinical T category (T2/3 vs. T4a) were performed. Within each stratum, multivariable Cox proportional hazards models were fitted with the same adjustment variables used in the primary cohort analysis. This ensured methodological consistency across all survival analyses. Within the T2/3 subgroup (n=50), there was no significant difference in LRFS survival between the MS and MP cohorts (aHR 0.42, 95% CI 0.04-4.85; p=0.489). Similarly, in the T4a subgroup (n=28), the MS cohort did not show an increased risk of LRFS compared to the MP cohort (aHR 0.55, 95% CI 0.0-6.45; p=0.632). The results were consistent for RRFS and DFS across both T stage strata (**Figure 4**).

## Discussion

This comparative cohort study demonstrates that for a carefully selected population with locally advanced OSCC abutting the mandible who achieved a rCR following NAT, a strategy of MP was associated with superior postoperative outcomes without compromising oncologic efficacy. Patients in the MP cohort experienced a significantly lower incidence of major complications, a markedly shorter hospital stay, and a substantially reduced burden of functional dependence compared to those undergoing MS. Longitudinal QoL assessments revealed a consistently superior and more rapid recovery across both global and head-and-neck-specific domains in the MP group. Most critically, with a median follow-up of three years, this organ-preserving approach did not result in inferior local, regional, or disease-free survival, providing preliminary evidence that the paradigm of mandatory mandibular sacrifice can be safely challenged in patients exhibiting an exceptional response to modern systemic therapy.

Our findings on surgical complication and functional recovery provide a stark, quantifiable illustration of the profound morbidity inherent to the conventional paradigm. The observed eight-fold higher rate of major postoperative complications in the MS cohort (19.4% vs. 2.4%) attests to the physiologic toll of segmental mandibulectomy and the requisite complex microvascular reconstruction. This drastic disparity is firmly rooted in established literature. As documented by van der Lely et al. (4) and corroborated by Nair et al. (3), the creation of a mandibular discontinuity defect is inherently associated with prolonged operative times, significant tissue trauma, and a high risk of sequelae such as flap failure and fistula formation, which aligns with the complications we observed predominantly in the MS group. Our results thus validate the known, substantial burden of composite resections.

The concept of using a biological checkpoint to guide the extent of surgery is not new, but our study demonstrates its renewed relevance in the era of effective systemic therapy. The seminal work by Wanebo et al. (10) established that surgical resection remains critical for maximizing tumor control after aggressive chemoradiation, particularly for patients with an incomplete pathologic response. Their protocol underscores the importance of a response-adapted approach. Our study builds upon this by demonstrating that for the subset achieving the most profound response, surgical de-escalation to a mandibular-preserving procedure is not only feasible but advantageous. Similarly, Deshpande et al. (11) reported that NAT led to tumor downstaging in tongue cancer, allowing a significant proportion of patients to undergo less morbid glossectomy with a high mandibular preservation rate. While their study focused on chemotherapy, it highlights the consistent theme that tumor response to neoadjuvant therapy creates an opportunity for organ preservation, a theme powerfully amplified in our cohort by the efficacy of immunotherapy.

Our findings are further substantiated by evidence questioning the necessity of radical mandibular resection. Stoop et al. (12) directly compared marginal versus segmental mandibulectomy for OSCC with confirmed bone invasion, finding no significant difference in locoregional recurrence or disease-specific survival, concluding marginal resection was oncologically safe when complete tumor removal was feasible. This provides a critical historical precedent, suggesting MP is not an inherently riskier strategy in appropriately selected patients, even without NAT. Our study extends this principle into the modern era, demonstrating that the exceptional responses elicited by NAT can safely expand the selection criteria for MP, allowing its application in more advanced cases that would have previously mandated segmental resection.

The stark contrast in functional outcomes further underscores the transformative impact of mandibular preservation. The preserved continuity of the mandibular arch in the MP cohort fundamentally underpinned a more physiologic and expedited recovery, translating into tangible clinical benefits: a markedly shorter median hospital stay (9 vs. 16 days) and dramatically lower rates of functional dependence. The significantly reduced reliance on gastrostomy tubes at discharge (28.6% vs. 63.9%) and the complete absence of tube dependence at 3 months in the MP group (0% vs. 11.1%) are particularly telling, attributable to the maintained structural integrity of the oral cavity crucial for mastication and deglutition. Similarly, the lower tracheostomy dependence at discharge (19.0% vs. 55.6%) highlights how MP avoids the glossoptosis and loss of anterior tongue support that often compromise the airway post-resection. This aligns with the principles in the phase II trial by Chaukar et al. (13). The collective evidence argues that in exceptional responders, an MP strategy successfully circumvents the extensive morbidity and protracted functional recovery long accepted as the trade-off for oncologic control in locally advanced OSCC involving the mandible (14).

The profound and clinically meaningful improvement in QoL observed in the MP cohort constitutes the most compelling argument for this de-escalation strategy. The longitudinal EORTC data revealed that the benefits of MP represented a substantial enhancement in patients’ lived experience, affecting core domains essential for reintegrating into normal life. Patients undergoing preservation demonstrated superior outcomes in speech, social eating, and social contact. The starkly lower scores for feeding tube dependence and pain medication use in the MP group translate into tangible, real-world benefits, such as reduced reliance on medical aids and a faster return to autonomy. This contrast underscores that sacrificing the mandible inflicts a severe and lasting deficit on a patient’s fundamental human functions, whereas preservation protects this functional integrity.

Our QoL findings are powerfully contextualized by morphometric and patient-reported outcomes in the literature. Diarra et al. (15) compared mandibular reconstructions using iliac versus fibular flaps. While they found no overall difference in global QoL, they identified critical domain-specific variations: fibular flaps were associated with better outcomes for pain and activity, whereas iliac flaps, which better preserve bone contour, scored higher in appearance and speech. This dichotomy highlights that even successful reconstruction cannot fully restore native form and function. The superior appearance and speech scores with the iliac flap indirectly support our direct finding that MP leads to the best possible QoL in these vital domains, without the associated donor-site morbidity and reconstructive risks.

Our findings powerfully align with and extend the growing evidence advocating for de-escalation. Fiedler et al. (16) identified chewing as the strongest impairment after fibula free flap reconstruction and highlighted that functional limitations like speech intelligibility correlated with poorer QoL. This directly corroborates our observation that the MS cohort suffered worse outcomes in these areas. Furthermore, Cao et al. (7) found that de-escalated surgery following neoadjuvant chemoimmunotherapy brought superior QoL. Our work confirms this principle and critically demonstrates its application in the high-stakes context of mandibular management, showing that the QoL benefit is not achieved at the cost of oncologic safety. By positioning MP as viable for patients achieving an rCR, we challenge the historical paradigm that equates radical resection with superior cancer control, offering a strategy that prioritizes both survival and its quality.

This paradigm shift towards response-adapted surgical de-escalation is gaining traction. Unlike other head and neck cancers where non-surgical organ-preservation is standard, OSCC management has remained predominantly surgical due to the perceived necessity of complete resection. This comes at a high functional cost. The emergence of effective NAT has challenged this, introducing the possibility of reducing surgical morbidity without compromising oncologic outcomes. Early trials hinted at the feasibility of a less aggressive approach (6). Subsequent research provided more direct evidence; Cao et al. (7) reported that most patients could avoid flap reconstruction and mandibulectomy after NAT with excellent survival, and Wu et al. (8) confirmed superior functional and QoL outcomes with NAT and limited surgery versus upfront surgery.

Our study builds upon and critically extends this evidence. While previous comparisons primarily contrasted NAT followed by surgery against upfront surgery, our investigation represents a pivotal, direct comparison within the modern treatment sequence: patients who achieved an rCR to NAT and were then treated with either MP or MS surgery. This design directly addresses the clinical dilemma faced after a major response. Our findings demonstrate that the MP approach confers significant benefits in safety, function, and QoL without incurring a penalty in local, regional, or disease-free survival over a median of three years. The improved outcomes are likely a direct result of preserving the native mandibular architecture and its surrounding soft tissue complex, maintaining the biomechanical integrity essential for mastication, deglutition, and speech. Consequently, our work provides robust evidence that for patients with OSCC exhibiting an exceptional response to NAT, mandibular preservation is not merely feasible but advantageous, successfully de-escalating surgery to enhance the patient’s postoperative experience and quality of life while maintaining oncologic control.

This study has several limitations. Its retrospective, non-randomized design introduces potential for selection bias, as the intraoperative decision to preserve the mandible was surgeon-assessed, creating cohorts with inherent differences. Notably, the MS cohort contained a higher proportion of patients with frank cortical bone erosion on pre-treatment imaging, reflecting the clinical tendency toward more radical resection in cases with evident bone involvement. This baseline imbalance in tumor invasiveness represents a major potential confounding factor that may have influenced both surgical outcomes and functional recovery. The modest sample size and low recurrence rate limit the statistical power for robust survival comparisons, resulting in wide confidence intervals. Furthermore, the median follow-up of three years is insufficient to evaluate long-term oncologic safety. These findings require validation through larger, prospective, multi-institutional trials with extended follow-up.

In conclusion, this study provides evidence supporting mandibular preservation as a potentially advantageous approach for carefully selected patients with locally advanced OSCC abutting the mandible who achieve a radiologic complete response following neoadjuvant immunotherapy. Compared to mandibular-sacrificing procedures, the preservation strategy was associated with a significantly superior postoperative safety profile along with a faster and more complete recovery of health-related quality of life. Importantly, within the limits of this retrospective analysis and over a median follow-up of three years, this surgical de-escalation did not appear to compromise short-to-medium-term oncologic control. Nevertheless, these conclusions should be interpreted cautiously, as they are derived from a non-randomized cohort with inherent selection bias. Further validation through prospective studies with appropriately matched groups and longer follow-up is needed before this approach can be widely adopted in clinical practice.

## Data Availability

The original contributions presented in the study are included in the article/**Supplementary Material**. Further inquiries can be directed to the corresponding author.
