# Supplementary material for: Mandibular preservation vs. sacrifice following neoadjuvant immunotherapy in locally advanced oral cancer: a comparative study of surgical and quality-of-life outcomes
Source: Front Oncol. 2026 Mar 4;16:1754661. doi: 10.3389/fonc.2026.1754661 (PMC12995778; doi:10.3389/fonc.2026.1754661)
Supplement: Supplementary file 1 [file Table1.doc]

### ****Supplementary Table 1: Baseline and Demographic Characteristics of the Mandibular Preservation (MP) and Mandibular Sacrificing (MS) Cohorts****

| **Characteristic** | **Overall (n=78)** | **MP Cohort (n=42)** | **MS Cohort (n=36)** | **p-value** |
| --- | --- | --- | --- | --- |
| ****Demographics**** |  |  |  |  |
| Age, years (Mean ± SD) | 58.5 ± 9.2 | 57.1 ± 8.8 | 60.2 ± 9.5 | 0.128 |
| Sex, Male (n, %) | 62 (79.5%) | 32 (76.2%) | 30 (83.3%) | 0.431 |
| BMI, kg/m² (Mean ± SD) | 23.1 ± 3.5 | 23.5 ± 3.2 | 22.6 ± 3.8 | 0.245 |
| Charlson Comorbidity Index (Median, IQR) | 3 (2-4) | 3 (2-4) | 4 (2-5) | 0.089 |
| ****Lifestyle Factors**** |  |  |  |  |
| Smoking History, >10 pack-years (n, %) | 45 (57.7%) | 22 (52.4%) | 23 (63.9%) | 0.302 |
| Alcohol Use, >7 units/week (n, %) | 38 (48.7%) | 18 (42.9%) | 20 (55.6%) | 0.257 |
| ****Tumor Characteristics**** |  |  |  |  |
| ****Bone Erosion (n, %)**** | 19 (24.4%) | 7 (16.7%) | 12 (33.3%) | 0.089 |
| ****Clinical T Stage (AJCC 8th)**** |  |  |  | ****0.008***** |
| T2 | 18 (23.1%) | 14 (33.3%) | 4 (11.1%) |  |
| T3 | 32 (41.0%) | 18 (42.9%) | 14 (38.9%) |  |
| T4a | 28 (35.9%) | 10 (23.8%) | 18 (50.0%) |  |
| ****Clinical N Stage (AJCC 8th)**** |  |  |  | 0.554 |
| N0 | 12 (15.4%) | 8 (19.0%) | 4 (11.1%) |  |
| N1 | 20 (25.6%) | 10 (23.8%) | 10 (27.8%) |  |
| N2 | 46 (59.0%) | 24 (57.1%) | 22 (61.1%) |  |
| ****Overall Stage Group**** |  |  |  | ****0.003***** |
| Stage III | 16 (20.5%) | 13 (31.0%) | 3 (8.3%) |  |
| Stage IVA | 49 (62.8%) | 25 (59.5%) | 24 (66.7%) |  |
| Stage IVB | 13 (16.7%) | 4 (9.5%) | 9 (25.0%) |  |
| **Postoperative therapy** |  |  |  | 0.834 |
| Radiotherapy | 38 (48.7%) | 20 (47.6%) | 18 (50.0%) |  |
| Chemoradiation | 40 (51.3%) | 22 (52.4%) | 18 (50.0%) |  |
| ****Neoadjuvant Treatment Details**** |  |  |  |  |
| ****NAT Cycles (Median, IQR)**** | 3 (2-4) | 3 (2-4) | 3 (2-4) | 0.711 |
| ****Any irAE (Grade ≥1)**** (n, %) | 35 (44.9%) | 18 (42.9%) | 17 (47.2%) | 0.692 |

*Data are presented as mean ± standard deviation, median (interquartile range), or number (percentage). p-values are from independent samples t-test, Mann-Whitney U test, or Chi-square/Fisher's exact test, as appropriate. Statistically significant values (p < 0.05) are in bold with an asterisk.*
Abbreviations: MP, Mandibular Preservation; MS, Mandibular Sacrificing; BMI, Body Mass Index; HPV, Human Papillomavirus; AJCC, American Joint Committee on Cancer; NAT, Neoadjuvant Therapy; ICI, Immune Checkpoint Inhibitor; irAE, immune-related Adverse Event.
