# Supplementary material for: Mandibular preservation vs. sacrifice following neoadjuvant immunotherapy in locally advanced oral cancer: a comparative study of surgical and quality-of-life outcomes
Source: Front Oncol. 2026 Mar 4;16:1754661. doi: 10.3389/fonc.2026.1754661 (PMC12995778; doi:10.3389/fonc.2026.1754661)
Supplement: Supplementary file 2 [file Table2.doc]

### ****Supplementary Table 2: Longitudinal Health-Related Quality of Life (EORTC QLQ-C30)****

| **Domain** | **Preoperation** |  | **3 months-post** |  | **6 months-post** |  | **12 months-post** |  |
| --- | --- | --- | --- | --- | --- | --- | --- | --- |
|  | ****MP**** | ****MS**** | ****MP**** | ****MS**** | ****MP**** | ****MS**** | ****MP**** | ****MS**** |
| ****Global QoL**** | 58.3 ± 12.5 | 56.0 ± 13.1 | 65.1 ± 11.8 | 58.5 ± 12.9 | ****78.2 ± 10.1**** | 66.8 ± 11.5 | ****84.5 ± 8.3**** | 75.2 ± 10.4 |
| ****Physical functioning**** | 75.6 ± 14.2 | 73.8 ± 15.0 | 72.4 ± 13.6 | 65.2 ± 14.8 | ****82.1 ± 11.2**** | 70.5 ± 13.1 | ****88.3 ± 9.1**** | 78.9 ± 11.8 |
| ****Role functioning**** | 70.2 ± 16.8 | 68.5 ± 17.2 | 68.8 ± 15.1 | 58.9 ± 16.5 | ****80.5 ± 12.4**** | 67.2 ± 14.9 | ****87.1 ± 10.5**** | 76.4 ± 13.2 |
| ****Emotional functioning**** | 68.9 ± 15.1 | 66.2 ± 16.0 | 75.6 ± 12.9 | 70.1 ± 14.2 | 81.3 ± 11.5 | 74.5 ± 13.8 | 85.7 ± 9.8 | 79.8 ± 12.1 |
| ****Cognitive functioning**** | 82.4 ± 10.5 | 80.1 ± 11.3 | 80.2 ± 11.1 | 76.5 ± 12.4 | 85.6 ± 9.8 | 79.8 ± 11.6 | 87.9 ± 8.7 | 82.1 ± 10.9 |
| ****Social functioning**** | 65.8 ± 17.5 | 63.4 ± 18.1 | ****70.5 ± 14.8**** | 60.2 ± 16.9 | ****83.4 ± 12.1**** | 68.9 ± 15.4 | ****89.2 ± 9.5**** | 77.6 ± 13.7 |
| ****Fatigue**** | 45.6 ± 18.2 | 48.2 ± 19.1 | 50.2 ± 16.5 | 58.9 ± 17.8 | ****35.8 ± 14.1**** | 45.6 ± 16.2 | ****28.4 ± 12.3**** | 36.7 ± 14.9 |
| ****Nausea/vomiting**** | 12.3 ± 8.5 | 14.1 ± 9.2 | 25.6 ± 10.2 | 30.5 ± 11.5 | ****10.2 ± 7.1**** | 18.7 ± 9.8 | 6.8 ± 5.9 | 12.3 ± 8.4 |
| ****Pain**** | 40.2 ± 16.8 | 42.5 ± 17.5 | ****35.6 ± 14.2**** | 50.8 ± 16.1 | ****22.1 ± 11.5**** | 35.4 ± 14.8 | ****15.3 ± 9.8**** | 24.6 ± 12.1 |
| ****Dyspnea**** | 15.4 ± 9.8 | 16.8 ± 10.5 | 18.9 ± 8.7 | 22.1 ± 9.9 | 12.3 ± 7.4 | 18.5 ± 9.1 | 10.1 ± 6.5 | 15.4 ± 8.2 |
| ****Insomnia**** | 35.6 ± 14.9 | 38.9 ± 15.7 | 40.2 ± 13.5 | 48.7 ± 14.8 | ****25.6 ± 11.2**** | 35.2 ± 13.6 | ****18.9 ± 9.8**** | 26.8 ± 12.1 |
| ****Appetite loss**** | 30.5 ± 12.5 | 32.1 ± 13.3 | ****38.9 ± 11.2**** | 52.3 ± 12.9 | ****20.1 ± 9.8**** | 35.6 ± 11.7 | ****12.4 ± 8.1**** | 22.1 ± 10.5 |
| ****Constipation**** | 18.9 ± 10.1 | 20.2 ± 10.8 | 22.1 ± 9.5 | 28.7 ± 10.9 | 15.4 ± 8.2 | 22.3 ± 9.8 | 10.5 ± 7.1 | 16.8 ± 8.9 |
| ****Diarrhea**** | 10.2 ± 6.8 | 11.5 ± 7.4 | 12.8 ± 6.1 | 15.6 ± 7.2 | 9.1 ± 5.5 | 13.4 ± 6.9 | 7.8 ± 4.9 | 11.2 ± 6.3 |
| ****Financial difficulty**** | 25.6 ± 11.2 | 28.9 ± 12.0 | 35.8 ± 10.5 | 45.6 ± 11.9 | 28.9 ± 9.1 | 38.7 ± 10.8 | ****20.1 ± 8.2**** | 30.5 ± 10.1 |

****Scoring Note:**** All scores are presented as mean ± standard deviation. For functioning and Global QoL scales, a higher score represents a better level of functioning or QoL. For symptom scales, a higher score represents a greater severity of symptoms. Bolded values indicate a statistically significant difference (p < 0.05) between MP and MS cohorts at that specific time point, based on linear mixed-effects model analysis.
****Abbreviations:**** MP, Mandibular Preservation; MS, Mandibular Sacrificing; SD, Standard Deviation.
