# Supplementary material for: Mandibular preservation vs. sacrifice following neoadjuvant immunotherapy in locally advanced oral cancer: a comparative study of surgical and quality-of-life outcomes
Source: Front Oncol. 2026 Mar 4;16:1754661. doi: 10.3389/fonc.2026.1754661 (PMC12995778; doi:10.3389/fonc.2026.1754661)
Supplement: Supplementary file 3 [file Table3.doc]

### ****Supplementary Table 3: Longitudinal Head and Neck-Specific Quality of Life (EORTC QLQ-H&N35)****

| **Domain** | **Preoperation** |  | **3 months-post** |  | **6 months-post** |  | **12 months-post** |  |
| --- | --- | --- | --- | --- | --- | --- | --- | --- |
|  | ****MP**** | ****MS**** | ****MP**** | ****MS**** | ****MP**** | ****MS**** | ****MP**** | ****MS**** |
| ****Pain**** | 45.2 ± 16.5 | 47.8 ± 17.2 | ****38.5 ± 14.1**** | 52.3 ± 16.8 | ****25.6 ± 11.8**** | 38.9 ± 15.1 | ****18.9 ± 9.5**** | 28.7 ± 12.4 |
| ****Swallowing**** | 35.8 ± 14.9 | 38.2 ± 15.7 | ****42.1 ± 13.5**** | 55.6 ± 16.2 | ****28.9 ± 11.2**** | 42.3 ± 14.8 | ****20.5 ± 9.1**** | 32.1 ± 12.6 |
| ****Senses**** | 25.6 ± 12.1 | 27.8 ± 12.9 | 28.9 ± 11.5 | 32.1 ± 13.2 | 22.1 ± 9.8 | 28.7 ± 12.1 | 18.9 ± 8.4 | 24.5 ± 10.8 |
| ****Speech**** | 32.1 ± 13.8 | 34.5 ± 14.6 | ****35.6 ± 12.4**** | 48.9 ± 15.1 | ****22.3 ± 10.5**** | 35.2 ± 13.9 | ****15.8 ± 8.7**** | 26.8 ± 12.1 |
| ****Social eating**** | 40.2 ± 15.8 | 42.9 ± 16.5 | ****45.6 ± 14.2**** | 58.7 ± 17.1 | ****30.5 ± 12.1**** | 45.1 ± 15.8 | ****22.1 ± 10.2**** | 35.6 ± 14.5 |
| ****Social contact**** | 28.9 ± 11.5 | 30.5 ± 12.3 | ****32.1 ± 10.8**** | 42.3 ± 13.5 | ****20.8 ± 9.1**** | 32.6 ± 12.4 | ****15.6 ± 7.8**** | 25.4 ± 11.2 |
| ****Sexual problems**** | 52.3 ± 18.2 | 54.1 ± 19.0 | 55.6 ± 16.8 | 58.9 ± 18.1 | 48.9 ± 15.1 | 52.3 ± 17.2 | 45.2 ± 13.8 | 48.7 ± 16.5 |
| ****Teeth**** | 38.7 ± 14.5 | 40.2 ± 15.3 | ****42.1 ± 13.2**** | 55.8 ± 16.1 | ****30.5 ± 11.5**** | 45.6 ± 14.9 | ****25.6 ± 10.1**** | 38.9 ± 13.8 |
| ****Open mouth**** | 35.6 ± 13.9 | 37.8 ± 14.7 | ****40.2 ± 12.5**** | 52.3 ± 15.8 | ****28.9 ± 10.8**** | 42.1 ± 14.2 | ****22.1 ± 9.5**** | 35.6 ± 12.9 |
| ****Dry mouth**** | 48.9 ± 16.8 | 50.5 ± 17.6 | 52.3 ± 15.5 | 58.7 ± 17.2 | 45.6 ± 13.9 | 52.1 ± 16.1 | 40.2 ± 12.5 | 48.9 ± 15.8 |
| ****Sticky saliva**** | 42.1 ± 15.2 | 44.5 ± 16.0 | 45.6 ± 14.1 | 52.3 ± 16.5 | 38.9 ± 12.8 | 48.7 ± 15.9 | 35.2 ± 11.5 | 42.1 ± 14.2 |
| ****Cough**** | 28.7 ± 11.8 | 30.2 ± 12.6 | 32.1 ± 10.9 | 35.6 ± 13.2 | 25.6 ± 9.8 | 30.5 ± 12.1 | 22.1 ± 8.5 | 28.7 ± 11.4 |
| ****Feeling ill**** | 35.2 ± 13.5 | 37.8 ± 14.3 | 38.9 ± 12.8 | 45.6 ± 15.1 | 28.7 ± 11.2 | 38.9 ± 14.5 | 22.3 ± 9.8 | 32.1 ± 13.2 |
| ****Pain medication**** | 22.1 ± 9.8 | 24.5 ± 10.5 | ****35.6 ± 11.2**** | 48.9 ± 13.8 | ****18.9 ± 8.5**** | 32.1 ± 12.1 | ****12.4 ± 7.1**** | 25.6 ± 10.9 |
| ****Food supplement**** | 18.9 ± 8.7 | 20.2 ± 9.4 | ****32.1 ± 10.5**** | 45.6 ± 12.9 | ****15.6 ± 7.8**** | 28.9 ± 11.5 | ****10.2 ± 6.5**** | 22.1 ± 10.2 |
| ****Feeding tube**** | 15.6 ± 7.5 | 16.8 ± 8.2 | ****28.9 ± 9.8**** | 42.1 ± 12.1 | ****12.3 ± 6.8**** | 25.6 ± 10.5 | ****8.9 ± 5.9**** | 18.9 ± 9.8 |
| ****Weight loss**** | 25.6 ± 10.8 | 27.8 ± 11.6 | ****35.2 ± 11.5**** | 48.7 ± 14.2 | ****20.1 ± 9.1**** | 35.6 ± 12.8 | ****15.6 ± 7.8**** | 28.9 ± 11.5 |
| ****Weight gain**** | 12.3 ± 6.8 | 13.5 ± 7.4 | 15.6 ± 6.2 | 18.9 ± 8.1 | 18.9 ± 7.1 | 22.1 ± 9.2 | 22.1 ± 8.5 | 25.6 ± 10.1 |

****Scoring Note:**** All scores are presented as mean ± standard deviation. For all symptom scales, a higher score represents a greater severity of symptoms. Bolded values indicate a statistically significant difference (p < 0.05) between MP and MS cohorts at that specific time point, based on linear mixed-effects model analysis.
****Abbreviations:**** MP, Mandibular Preservation; MS, Mandibular Sacrificing; SD, Standard Deviation
