# Supplementary material for: Mandibular preservation vs. sacrifice following neoadjuvant immunotherapy in locally advanced oral cancer: a comparative study of surgical and quality-of-life outcomes
Source: Front Oncol. 2026 Mar 4;16:1754661. doi: 10.3389/fonc.2026.1754661 (PMC12995778; doi:10.3389/fonc.2026.1754661)
Supplement: Supplementary file 4 [file Table4.doc]

### ****Supplementary Table 4: Univariate and Multivariable Cox Regression Analysis for Regional Recurrence-Free Survival (RRFS)****

| Factor | Univariate Analysis | | | Multivariable Analysis | | |
| --- | --- | --- | --- | --- | --- | --- |
|  | ****HR**** | ****95% CI**** | ****p**** | ****Adjusted HR**** | ****95% CI**** | ****p**** |
| ****Treatment Cohort**** (Ref: MP) |  |  |  |  |  |  |
| MS Cohort | 1.80 | 0.53-6.15 | 0.350 | 1.92 | 0.55-6.76 | 0.305 |
| ****Age**** (≥60 vs. <60 years) | 1.25 | 0.28-5.55 | 0.770 | 1.10 | 0.24-5.12 | 0.902 |
| ****Sex**** (Male vs. Female) | 1.08 | 0.24-4.79 | 0.920 | 0.95 | 0.20-4.57 | 0.945 |
| ****CCI**** (≥3 vs. <3) | 1.65 | 0.37-7.35 | 0.512 | 1.45 | 0.31-6.77 | 0.637 |
| ****Smoking**** (>10 vs. ≤10 pack-years) | 1.48 | 0.33-6.61 | 0.611 | 1.35 | 0.29-6.23 | 0.700 |
| ****Clinical T Stage**** (Ref: T2-T3) |  |  |  |  |  |  |
| T4a | 2.42 | 0.57-10.3 | 0.233 | 2.25 | 0.51-9.97 | 0.283 |
| ****Clinical N Stage**** (N2/3 vs. N0/1) | 1.55 | 0.36-6.65 | 0.556 | 1.40 | 0.32-6.20 | 0.652 |
| ****NAT Cycles**** (>2 vs. 2) | 1.21 | 0.28-5.21 | 0.798 | 1.15 | 0.26-5.11 | 0.854 |
| ****irAE**** (Yes vs. No) | 0.75 | 0.17-3.26 | 0.699 | 0.78 | 0.17-3.55 | 0.744 |

****Abbreviations:**** CI = Confidence Interval; HR = Hazard Ratio; MP = Mandibular Preservation; MS = Mandibular Sacrificing; CCI = Charlson Comorbidity Index; NAT = Neoadjuvant Therapy; irAE = immune-related Adverse Event.
