# Supplementary material for: Mandibular preservation vs. sacrifice following neoadjuvant immunotherapy in locally advanced oral cancer: a comparative study of surgical and quality-of-life outcomes
Source: Front Oncol. 2026 Mar 4;16:1754661. doi: 10.3389/fonc.2026.1754661 (PMC12995778; doi:10.3389/fonc.2026.1754661)
Supplement: Supplementary file 5 [file Table5.doc]

### ****Supplementary Table 5: Univariate and Multivariable Cox Regression Analysis for Disease-Free Survival (DFS)****

| Factor | Univariate Analysis | | | Multivariable Analysis | | |
| --- | --- | --- | --- | --- | --- | --- |
|  | ****HR**** | ****95% CI**** | ****p**** | ****Adjusted HR**** | ****95% CI**** | ****p**** |
| ****Treatment Cohort**** (Ref: MP) |  |  |  |  |  |  |
| MS Cohort | 0.35 | 0.04 - 3.18 | 0.350 | 0.33 | 0.03 - 3.12 | 0.332 |
| ****Age**** (≥60 vs. <60 years) | 1.45 | 0.24 - 8.69 | 0.685 | 1.32 | 0.21 - 8.18 | 0.767 |
| ****Sex**** (Male vs. Female) | 1.12 | 0.18 - 6.83 | 0.902 | 0.95 | 0.15 - 5.98 | 0.956 |
| ****CCI**** (≥3 vs. <3) | 1.80 | 0.30 - 10.78 | 0.520 | 1.65 | 0.26 - 10.29 | 0.594 |
| ****Smoking**** (>10 vs. ≤10 pack-years) | 1.52 | 0.25 - 9.10 | 0.649 | 1.48 | 0.24 - 9.05 | 0.671 |
| ****Clinical T Stage**** (Ref: T2-T3) |  |  |  |  |  |  |
| T4a | 2.67 | 0.45 - 15.71 | 0.279 | 2.10 | 0.34 - 12.85 | 0.417 |
| ****Clinical N Stage**** (N2/3 vs. N0/1) | 1.63 | 0.27 - 9.71 | 0.590 | 1.45 | 0.24 - 8.69 | 0.685 |
| ****NAT Cycles**** (>2 vs. 2) | 1.33 | 0.22 - 7.89 | 0.756 | 1.25 | 0.21 - 7.45 | 0.806 |
| ****irAE**** (Yes vs. No) | 0.79 | 0.13 - 4.70 | 0.797 | 0.85 | 0.14 - 5.12 | 0.861 |

CI = Confidence Interval; HR = Hazard Ratio; MP = Mandibular Preservation; MS = Mandibular Sacrificing; CCI = Charlson Comorbidity Index; NAT = Neoadjuvant Therapy; irAE = immune-related Adverse Event.
